# Annual of the Universal Medical Sciences

**Published:** 1891-10

**Authors:** 


ANNUAL OF THE UNIVERSAL MEDICAL SCIENCES. A Yearly Report of the Progress of the General Sanitary Sciences throughout the World. Edited by CHARLES E. SAJOUS, M. D., and Seventy Associate Editors, assisted by over Two Hundred Corresponding Editors, Collaborators and Correspondents. Illustrated with chromo-lithographs, engravings and maps. Five volumes. Philadelphia: F. A. Davis. 1891.

The fourth series of this invaluable work appears in its usual handsome form, and with all its wonted vigor and excellence.

Though published slightly later than usual, it is none the less welcome, nor diminished in usefulness. The only wonder is that the accomplished editor, indefatigable worker though he is, can bring out this stupendous work with such regularity and perfection as have characterized the four issues that are now in the hands of the profession. The slight delay in the present series has been due to causes that must ever menace all human enterprises—sickness and death. Two of the editor's three confidential secretaries were thus removed from active participation in the preparation of these volumes—one by grievous illness, prolonged in character, and the other fell a victim to fatal pneumonia. To the remaining faithful aid, Miss N. I. McCarthy—we gladly mention her name—the editor pays a graceful tribute in his preface.

The present series bears abundant evidence of the wisdom of the projector and editor of the *Annual*, in its every volume, department, sсction and page, and it has become a necessity for teachers, practitioners, authors, and students of medical literature. None of these can afford to do without it, and many other scientific workers ought to possess it. It has become a part of the essential periodical literature of American medicine, and will, no doubt, continne to appear during the future years on much the same lines that at present mark its unexampled prosperity. We have nothing but praise for both editor and publisher in giving to the profession such a beautiful and useful work. It should be encouraged by a liberal patronage in all lands.

